# Inhibition of the Differentiation of Monocyte-Derived Dendritic Cells by Human Gingival Fibroblasts

**DOI:** 10.1371/journal.pone.0070937

**Published:** 2013-08-02

**Authors:** Sylvie Séguier, Eric Tartour, Coralie Guérin, Ludovic Couty, Mathilde Lemitre, Laetitia Lallement, Marysette Folliguet, Samah El Naderi, Magali Terme, Cécile Badoual, Antoine Lafont, Bernard Coulomb

**Affiliations:** 1 Inserm U970 Team Laboratory of Arterial Repair, Université Paris Descartes, Sorbonne Paris Cité, PARCC/Hopital Européen Georges Pompidou, Paris, France; 2 Université Paris Descartes, Sorbonne Paris Cité, Faculté d’Odontologie, Montrouge, France; 3 Service d’Odontologie, Hôpital Louis Mourier, Colombes, France; 4 Inserm U970 Team Immunotherapy and anti-angiogenic therapy in oncology, Université Paris Descartes, PARCC/HEGP, Paris, France; 5 Flow Cytometry Core Facility, Inserm U970, Université Paris Descartes, PARCC/HEGP, Paris, France; Institut National de la Santé et de la Recherche Médicale U 872, France

## Abstract

We investigated whether gingival fibroblasts (GFs) can modulate the differentiation and/or maturation of monocyte-derived dendritic cells (DCs) and analyzed soluble factors that may be involved in this immune modulation. Experiments were performed using human monocytes in co-culture with human GFs in Transwell® chambers or using monocyte cultures treated with conditioned media (CM) from GFs of four donors. The four CM and supernatants from cell culture were assayed by ELISA for cytokines involved in the differentiation of dendritic cells, such as IL-6, VEGF, TGFβ1, IL-13 and IL-10. The maturation of monocyte-derived DCs induced by LPS in presence of CM was also studied. Cell surface phenotype markers were analyzed by flow cytometry. In co-cultures, GFs inhibited the differentiation of monocyte-derived DCs and the strength of this blockade correlated with the GF/monocyte ratio. Conditioned media from GFs showed similar effects, suggesting the involvement of soluble factors produced by GFs. This inhibition was associated with a lower stimulatory activity in MLR of DCs generated with GFs or its CM. Neutralizing antibodies against IL-6 and VEGF significantly (P<0.05) inhibited the inhibitory effect of CM on the differentiation of monocytes-derived DCs and in a dose dependent manner. Our data suggest that IL-6 is the main factor responsible for the inhibition of DCs differentiation mediated by GFs but that VEGF is also involved and constitutes an additional mechanism.

## Introduction

Fibroblasts, the most abundant cells of the stroma, are characterized by their morphology, ability to adhere, their production and degradation of the extracellular matrix (ECM) and the absence of epithelial, vascular and leukocyte lineage markers. Gingival fibroblasts (GFs) are involved in tissue remodeling of the oral mucosa and contribute to the rapid healing of oral wounds without scarring in the gingiva. Remodeling of tissue during wound repair requires controlled synthesis and degradation of ECM and resolution of inflammation [1]. The details of the immunomodulatory properties of human GFs and their role in the maintenance and initiation of inflammatory disease are still unclear. Nevertheless, in rheumatoid arthritis, fibroblasts modify the quality, intensity and duration of the inflammatory infiltrate during the induction of inflammatory responses [2]. IFN-gamma-treated GFs inhibit the proliferative responses of phytohemagglutinin (PHA)-stimulated T cells [3]. Fibroblasts have a direct role in suppressing immune responses in the spleen, where they drive the development of regulatory dendritic cells (DCs), following their activation by infectious agents [4]. Dermal fibroblasts release IL-6, which up-regulates the expression of functional M-CSF receptors on monocytes, allowing the monocytes to bind autocrine M-CSF [5]–[7] which switches monocyte differentiation to macrophages rather than DCs. A recent study showed that human cytomegalovirus induced production of IL-6 by infected cells leading to the inhibition of DC differentiation [8]. DCs, the most potent antigen-presenting cells (APCs), can be generated from blood monocytes *in vitro* in the presence of recombinant granulocyte-macrophage colony-stimulating factor (rGM-CSF) and recombinant interleukin-4 (rIL-4) [9]; however, myeloid differentiation is complex, not fully understood, and influenced by various factors. DCs play a major role in the uptake, transport, and presentation of antigens and display the unique capacity to stimulate naïve T lymphocytes [10]. The initiation of immune responses is associated with the differentiation and the maturation of DCs and their migration to draining lymph nodes. Thus, immune cells and their progenitors encounter cells of the tissue microenvironment, including fibroblasts. Several studies have reported the effects of stromal cells on the regulation of DC functions in the normal healthy state as well as in inflammatory conditions [11], [12]. Fibroblasts may be a major source of anti-inflammatory mediators and are thought to be involved in the regulation of DC functions: indeed, they synthesize factors modulating DC functions, such as chemokines and other cytokines (including IL-6 and TGFβ), matrix components and matrix-degrading enzymes [13]. Therapeutic utilization of fibroblasts and their biologically active products is an emerging approach for the control of chronic inflammatory diseases [14].

To our knowledge, the effect of GFs on the differentiation of DCs has not been rigorously described and the demonstration that adult GFs can modulate early stages of DC differentiation would have important implications for local immunity in the gingiva. In this work, we show that human GFs *in vitro* actively participate in the local regulation of the immune response through the secretion of IL-6 and VEGF and thereby their capacity to inhibit the differentiation of monocyte-derived dendritic cells.

## Materials and Methods

### Human Gingival Fibroblasts (GFs) and Conditioned Medium from GF Culture (CM)

Healthy gingival tissue samples which would otherwise have been discarded were obtained from healthy patients undergoing tooth extraction of the third molar or orthodontic procedures. The study was approved by the local ethics committee (Comité de Protection des Personnes (CPP) “Ile de France II” (May 11^th^ 2012), IRB registration: 00001072) and all subjects gave written informed consent. The patients included in this study (n = 10, 5 females and 5 males, aged 20–40) had neither other oral or systemic diseases, nor any overt immunological abnormalities and did not take any preoperatory medication. For immunofluorescence staining, the tissue samples were immediately frozen and stored at −80°C. Primary explant cultures were established in 25 cm^2^ culture flasks in RPMI (Gibco, France) containing 10% fetal bovine serum (FBS), penicillin (100 µg/mL), streptomycin (100 µg/mL) and fungizone (2 ng/mL). GFs expanded in culture showed positive surface staining for CD90 and were negative for CD14, CD31 and CD45. Monolayer cultures were maintained under a humid atmosphere of 5% CO_2,_ 95% air at 37°C. When the cultures reached confluence, adherent cells were detached with 0.25% trypsin and reseeded for expansion (passage) in 75 cm^2^ culture flasks. Conditioned media (CM) from GFs grown to confluence in one week was collected from passage 3 and filtered through 0.22 µm pore-sized filters (Corning). The absence of toxicity of the CM for monocytes and the reproducibility between fresh and once-frozen CM was verified in preliminary experiments (not shown). Experiments were performed using once-frozen CM. CM from four independent GF donors were used. CM and GFs were tested for mycoplasma contamination (PlasmoTest, Invivogen, San Diego, USA).

### Human CD14^+^ Monocytes, Differentiation and Maturation of Monocyte-derived Cells

Human monocytes (n = 25 donors, more than 90% CD14^+^, provided by CIC BT Nantes, France) were cultured in RPMI 1640 containing 10% fetal bovine serum (FBS), penicillin (100 µg/mL), streptomycin (100 µg/mL) and fungizone (2 ng/mL). The differentiation of monocytes was induced by a mixture of recombinant GM-CSF (rGM-CSF, CellGenix, Germany, 1000 U/mL) and recombinant IL-4 (rIL-4, CellGenix, Germany, 200 U/mL), referred to as medium for differentiation (MD), for 7 days. Transwell® chambers, with a 0.4 µm pore-size membrane (Corning) which prevents cell-cell contact, were used to investigate the effects of GFs on the differentiation of DCs. At day 0, non confluent fibroblasts (2×10^5^ cells per well) were seeded in the lower compartment and monocytes (3×10^6^ cells) were seeded on the upper compartment. After 7 days of coculture, the GFs formed a confluent layer, and the viability of fibroblasts and monocytes was assessed (n = 5 experiments in duplicate) by double staining with PI (Invitrogen) and FITC Annexin V (Biolegend). Various fibroblast/monocyte ratios were tested (1∶5, 1∶10, 1∶20 and 1∶25). In five experiments, we also used dermal fibroblasts (DFs) from human skin (DF/monocyte ratio 1∶25) to compare the effects of GFs and DFs on the differentiation of monocyte-derived dendritic cells. DFs were also tested for mycoplasma contamination (PlasmoTest, Invivogen, San Diego, USA).

The effect of conditioned media (CM, 2.5 mL/well) from GF culture was analyzed using 6-well flat-bottomed plates: each well contained 5 mL of whole media and was seeded with 2×10^6^ monocytes. In additional experiments, maturation of monocyte-derived cells was induced by adding lipopolysaccharide (LPS, *Salmonella typhosa*; Sigma-Aldrich) (5 µg/mL) on day 7 and cells were analyzed 48 h later. Monocyte-derived cell morphology was analyzed on cytospin slides prepared by centrifuging 50,000 cells onto glass microscope slides. These slides were dried, fixed with methanol, stained with Giemsa stain, and examined under a Axioscop 20 microscope (Zeiss, Germany) equipped with Achroplan ×5, ×10, and ×20 objective lenses (Zeiss, Germany). Images were captured with a Spot insight FireWire camera (Diagnostic instrument, USA) and processed with IPS 4.02 software (Tribvn, France).

### Analysis of Cell Surface Phenotype Markers by Flow Cytometry

Monocyte-derived cells were labeled with PE anti-human CD1a (HI149, BioLegend, USA), APC anti-human DC-Sign (9E9A8, BioLegend, USA), Pacific Blue anti-human CD14 (HCD14, BioLegend, USA), APC anti-human CD80 (2D10, BioLegend, USA), FITC anti-human CD83 (HB15e, BioLegend, USA), Pacific Blue anti-human CD86 (IT2.2, BioLegend, USA) and FITC anti-human HLA-DR (Immu-357, Beckman Coulter, France). Cells were counted and analyzed on a LSRII Flow Cytometer (Becton Dickinson) with FacsDiva software (Becton Dickinson). Appropriate isotypic control antibodies were included in each analysis, with acquisition and analysis gates set accordingly. FlowJo software (Treestar) was used for data analysis. Results are expressed as percentages of positive cells.

### Allogeneic Mixed Lymphocyte Reaction

Monocyte-derived DCs obtained after 7 days of induction with rGM-CSF and rIL-4 with or without CM from GFs were stimulated with LPS for an additional 48 hours to promote the maturation of DCs. Mature DCs obtained with or without CM were used to stimulate 2×10^5^ purified allogeneic CD4^+^T cells (DC/T ratio was 1/8) purified from PBMCs from two donors. After 4 days, cells were pulsed overnight with 1 µCi of ^3^H-thymidine to determine T cell proliferation.

### Detection of Cytokines in Conditioned Media from Gingival Fibroblasts (GFs) by Enzyme-linked Immunosorbent Assay (ELISA)

The four conditioned media (CM) from the GFs from four donors were tested for IL-6, VEGF, TGFβ1 (R&D systems, France), IL-13 (Abcam, France) and IL-10 (Diaclone, France) by ELISA according to the instructions of the kit manufacturers. The supernatants from samples of monocytes cultured for 7 days in MD (rGM-CSF and rIL-4) in the presence or absence of CM were similarly assayed for these cytokines. CM incubated for 7 days in the same conditions as monocyte cultures were also tested to verify its stability.

### Neutralizing Antibodies against IL-6, VEGF and TGFβ1

In some experiments, neutralizing antibodies against VEGF (1 µg/mL and 10 µg/mL), TGFβ (1 µg/mL) and IL-6 (1 µg/mL and 10 µg/mL) (R&D Systems, France) were added to CM before its addition to monocyte cultures to analyze their effects on DC differentiation. Appropriate isotypic controls (rabbit IgG antibody or mouse IgG1 antibody, R&D Systems, France) at the same concentrations were included in each experiment.

### Differentiation of Monocyte-derived Dendritic Cells in the Presence of Recombinant VEGF (rVEGF)

To study the effect of VEGF on monocyte differentiation, we also performed experiments involving addition of rVEGF (10 ng/mL, 20 ng/mL, 40 ng/mL and 80 ng/mL) to monocytes cultured with rIL-4 and rGM-CSF.

### Detection of IL-6 in Gingival Biopsies by ELISA and RT-PCR

Total proteins were extracted from 10–20 mg human gingival biopsies (n = 3) using the Mammalian cell lysis kit (Sigma, Mi, USA) and IL-6 analysis was performed using IL-6 enzyme-linked immunosorbent assay kits (ELISA) (R&D Systems, France).

Total RNA was extracted from 10–20 mg samples of human gingiva biopsies (n = 3) using the Qiagen RNeasy kit for extraction from fibrous tissue (Qiagen, Courtaboeuf, France). The total RNA concentration in each preparation was determined, in triplicate, from the 260 nm/280 nm absorbance ratio as measured with a photometer (BioPhotometer plus, Eppendorph, Le Pecq, France). Human IL-6 mRNA was assayed in 400 ng aliquots of total RNA for each condition as follows: the Biorad Iscrpit cDNA synthesis kit (Biorad, Marnes-la-Coquette, France) was used to produce cDNA, and iQ SYBR Green Supermix (Biorad, Marnes-la-Coquette, France) and Human IL-6 primers (Qiagen Quantitect primer QT00083720 Qiagen, Courtaboeuf, France) were for quantitative PCR in a CFX-96 system with human GAPDH as the reference. Each PCR was repeated three times in duplicate and data were analyzed with CFX manager Software (Biorad, Marnes-la-Coquette, France) by the delta CT method.

### Co-localization of Gingival Fibroblasts (GFs) and Monocytes by Immunohistochemistry

Frozen specimens of gingiva (n = 3) were cut into 4–6 µm-thick sections with a cryostat, and the sections placed on slides, air dried and fixed for 10 minutes with 100% acetone. The slides were treated with an avidin/biotin blocker (Vector Laboratories, Burlingame USA) and the Fc receptor was blocked by human serum (5%). Then, antibodies diluted in PBS were added: biotin anti-human CD90 was used at a dilution of 1/500 (5E10 Biolegend, San Diego, USA) to recognize GFs and FITC anti-human CD14 at 1/300 (M5E2 Biolegend San Diego, USA) to label monocytes. Isotype-matched antibodies were used as negative controls. In each case, we checked that the secondary antibodies did not cross-react with the isotype or species of the other primary antibody used for double immunofluorescence labeling. Fluorescent images of mounted sections were acquired with an epifluorescence microscope DMR (Leica Microsystems, Wetzlar, Germany).

### Statistics

Results are expressed as means ± SD for at least three independent experiments in duplicate. Statistical significance was determined by Student’s t-test (paired-data analysis). P values <0.05 were considered as statistically significant.

## Results

### Monocyte-derived Cell Morphology is Affected by the Presence of Gingival Fibroblasts (GFs) or Conditioned Medium from GFs

**Figure 1:**
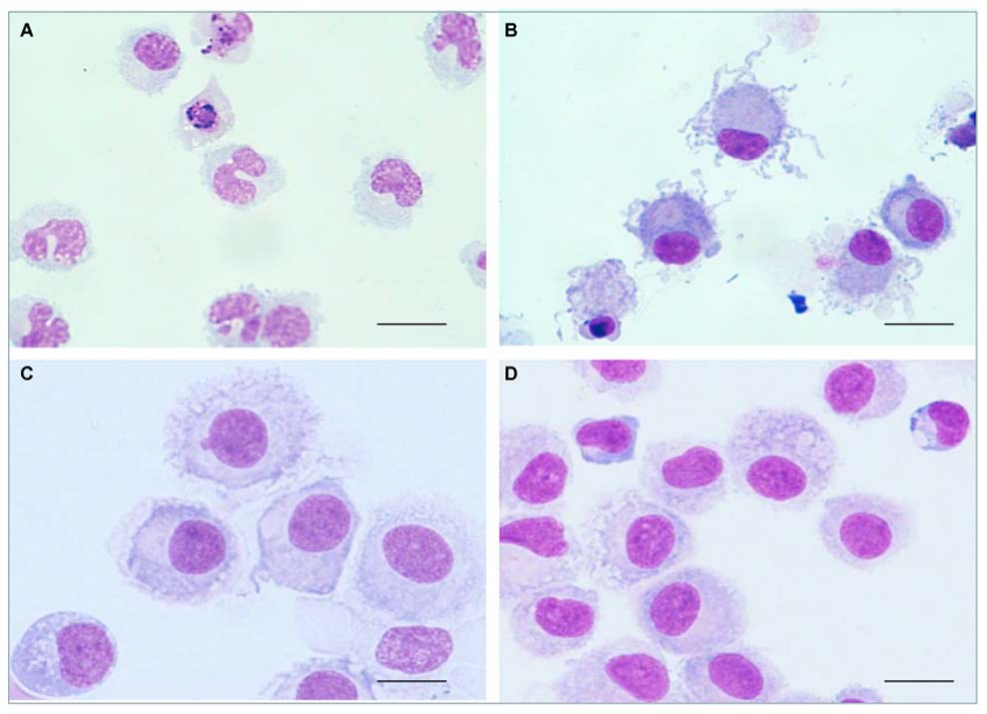
Gingival fibroblasts (GFs) or conditioned medium from GFs modify the morphology of monocyte-derived dendritic cells. Giemsa staining on cytospin slides of cells prepared from cultures of A. Monocytes (day 0). B. Monocyte-derived dendritic cells after 7 days in culture with medium for differentiation (MD, containing rGM-CSF and rIL-4); these cells present the typical characteristics of DC with clearly visible dendrites. C. Monocyte-derived cells co-cultured for 7 days with GFs in a Transwell® system with MD. D. Monocyte-derived cells cultured for 7 days in the presence of conditioned medium from GFs with MD. Monocyte-derived cells (C and D) and monocyte-dendritic cells (B) show morphological differences, in particular the absence of typical dendritesfrom monocyte-derived cells. The results are representative of three independent experiments. Scale bar: 20 µm, original magnification ×400.

As expected, CD14^+^ monocytes (Fig. 1A) cultured with MD (rGM-CSF and rIL-4) for 7 days acquired the size and protruding veils typical of the morphology of DCs (Figure 1B). Monocytes cultured in the Transwell® apparatus (Fig. 1C) with gingival fibroblasts (GFs), such that the two cell types were separated by a porous membrane, or in presence of conditioned medium (CM) (Fig. 1D), were round, and did not develop the veiled appearance or other morphological characteristics (dendrites) of DCs. This indicates that factors secreted by GFs modify the process of differentiation of monocyte-derived DCs.

### Soluble Factors from Fibroblasts Inhibit the Differentiation but not the Maturation of Monocyte-derived Dendritic Cells

**Figure 2:**
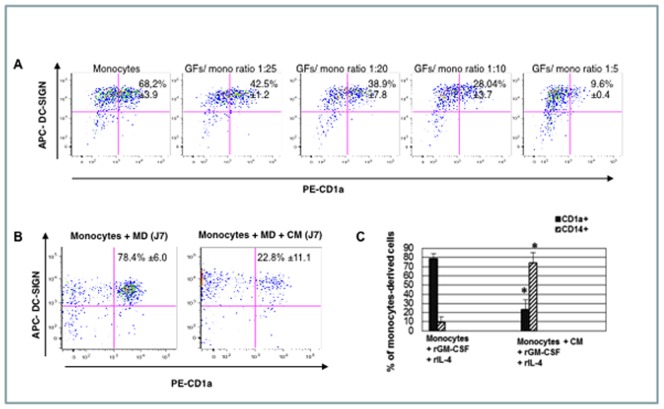
Soluble factors from gingival fibroblasts (GFs) inhibit the differentiation of CD1a^+^ dendritic cells. A. Analysis of the induction of the differentiation of monocyte-derived DCs by MD (rGM-CSF and rIL-4) after 7 days co co-culture of GFs (lower compartment) with monocytes (upper compartment) in a Transwell® chamber system. The effect of several GF/monocyte ratios (1∶25 to 1∶5) on CD1a and DC-SIGN expression was analyzed by flow cytometry. Results are expressed as percentages ± SD of CD1a^+^ DC-SIGN^+^ positive cells in the upper right part of each figure. Percentages of CD1a^+^ DC-SIGN^+^ dendritic cells decreased with increasing GF/monocyte ratio. Percentages are means ± SD of three independent experiments. B. Monocytes cultured with conditioned medium (CM) from GFs in the presence of MD for 7 days. Results are representative of four separate experiments and results are expressed as percentages ± SD of CD1a^+^ DC-SIGN^+^ positive cells in the upper right part of each figure. C. The differentiation of monocyte-derived DCs was also monitored by assessing the expression of CD1a and CD14 on day 7. The percentage of CD1a^+^ dendritic cells was significantly lower (P<0.0001) and that of CD14^+^ cells significantly higher (P<0.0001) in the presence of CM than in controls. Results are expressed as means ± SD and the histogram reports the results of 12 independent experiments using monocytes from 12 different donors. CM: conditioned medium. * Significant difference (P<0.0001).

The inhibition of the differentiation of monocyte-derived DCs cultured in MD increased with the GF/monocyte ratio (1∶25 to 1∶5). Thus, the effect of GFs was dose dependent, appeared to be mediated, at least in part, by soluble factors (Figure 2A). DC differentiation was inhibited by both gingival (GFs) and dermal fibroblasts (DFs) in the Transwell® apparatus, and at the same fibroblast/monocyte ratio (1∶25) the difference between the effects of GFs and DFs was not significant (46.2% CD1a^+^ cells ±1.3 for DFs versus 43% ±1.5 for GFs). On D7, the mean percentage of PI+ annexin V+ monocytes was 10.22% ±2.54 and that of PI+ annexin V+ fibroblasts was 19.37% ±7.95 (data not shown).

In 12 independent experiments using monocytes from 12 different donors, conditioned medium (CM) from GFs significantly inhibited the differentiation of CD1a^+^ monocyte-derived DCs (Figures 2B and 2C). The inhibition of the differentiation of CD1a^+^ monocytes-derived DCs was statistically significant for four independent preparations of CM from GFs (Table 1). GFs from all gingival biopsies were positive for mycoplasma, which is physiologically present in the oral cavity [15], [16]. However, DFs were negative for mycoplasma and CM from DFs significantly inhibited the differentiation of CD1a^+^ monocyte-derived DCs (68.75% ±11.16 CD1a+ cells versus 13.66% ±8.98 in presence of CM from DFs, P<0.0001). The effect of LPS was evidenced by an increase of the Mean Fluorescence Intensity (MFI) following labeling with antibodies against CD80, CD83, CD86 and HLA-DR: CM from GFs did not inhibit or increase the effects of LPS (data not shown).

**Table 1:** Percentage of the differentiation of CD1a+ monocyte-derived dendritic cells using conditioned media from 4 independent gingival fibroblasts donors.

	CD1a+ monocyte-deriveddendritic cells
Monocytes+MD	77.9% ±7.89
Monocytes+MD+**CM1**	19.7% ±7.79*
Monocytes+MD	71.9% ±12.54
Monocytes+MD+**CM2**	28.2% ±10.47*
Monocytes+MD	62.3% ±9.03
Monocytes+MD+**CM3**	27.7% ±8.07*
Monocytes+MD	87.1% ±0.2
Monocytes+MD+**CM4**	52.1% ±3.8*

MD: medium for differentiation (rGM-CSF+rIL-4).

CM1, CM2, CM3 and CM4: conditioned media from independent gingival fibroblasts donors 1 to 4.

For each CM, results are shown as mean ± SD of at least 3 independent experiments in duplicate.

* P<0.0001.

### Allogeneic Mixed Lymphocyte Reaction (MLR)

To assess whether the inhibition of DC differentiation resulted in these cells being less able to stimulate T cells, we performed a MLR using DCs generated in various conditions.

**Figure 3:**
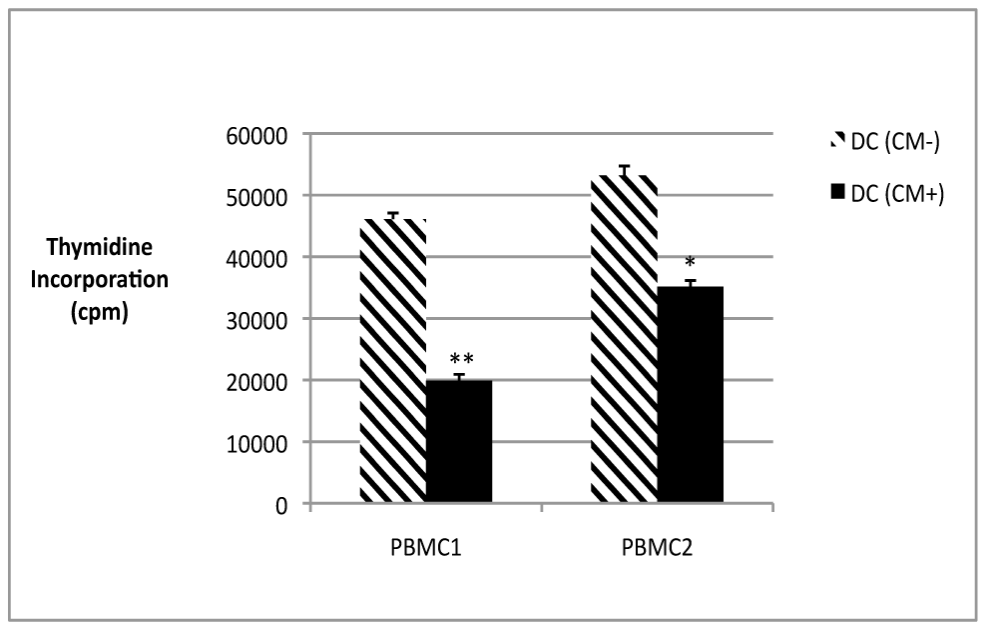
Mature monocyte-derived DCs obtained with (CM+) or without (CM-) conditioned medium from GFs were used to stimulate allogeneic CD4+ T cells purified from the PBMCs from two donors (PBMC1 and PBMC2); thymidine incorporation is expressed in counts per minute (cpm). Mature monocyte-derived DCs obtained in the presence of CM from GFs displayed a significantly capacity than controls to stimulate the proliferation of allogeneic CD4+T cells. Mean ± SD of triplicates are shown and results are representative of two independent experiments. *Significant difference (P = 0.002) and **Significant difference (P<0.001).

Mature monocyte-derived dendritic cells obtained in the presence of CM from GFs displayed a significantly lower capacity than controls to stimulate the proliferation of allogeneic CD4^+^T cells purified from PBMCs from two donors: the mean percentage of inhibition in the presence of CM was 44% of the control value (P<0.001 and P = 0.002, respectively) (Figure 3).

### VEGF, IL-6 and TGFβ1 Production by GFs

We tested CM from GF cultures for factors known to be involved in inhibitory effects on DC differentiation (Table 2).

**Table 2:** Detection of IL-6, VEGF and TGFβ1 by ELISA in CM and supernatant from cell culture (pg/mL).

	IL-6	VEGF	TGFβ1
**CM**	2728.7±137.2	2406.2±362.4	2602.5±432.6
**CM (D7)**	2542.5±84.2	2174.7±318.3	2368.7±369.1
**Monocytes with MD (D7)**	162.3±6.6	ND	1312.4±394.1
**Monocytes with MD and CM (D7)**	1682.2±172.2	ND	2476.4±285.7

Results are representative of 4 independent experiments realized in duplicate.

IL-13 and IL-10 were not detected in CM and supernatant from cell culture.

MD: medium for differentiation (rGM-CSF+rIL-4).

CM: conditioned medium from gingival fibroblasts.

D7: after 7 days in culture.

ND: not detected.

The means of concentrations ± SD detected by ELISA in the four CM were 2728.7 pg/mL ±137.2 for IL-6, 2406.2 pg/mL ±362.4 for VEGF and 2602.5 pg/mL ±432.6 for TGFβ1. IL-13 and IL-10 were not detected by ELISA in any of the four CM from GFs from four independent donors. These analyses also confirmed the stability of CM kept for 7 days in the same conditions as monocyte cultures. VEGF completely disappeared in presence of monocytes, presumably due to its internalization following binding to the VEGF-R expressed by these cells (Table 2).

Monocytes cultured for 7 days in the MD (rGM-CSF and rIL-4) released small amounts of IL-6 and more TGFβ. The production of IL-6 or TGFβ by monocytes was not higher in the presence of CM than in CM alone (Table 2). GFs from all gingival biopsies were positive for mycoplasma and this may explain the high levels of IL-6 in CM. However, all CM from gingival fibroblasts were negative for mycoplasma contamination.

### Neutralization of IL-6 and VEGF Partially Abolishes the Inhibitory Effect of GF and its CM on DC Differentiation

**Figure 4:**
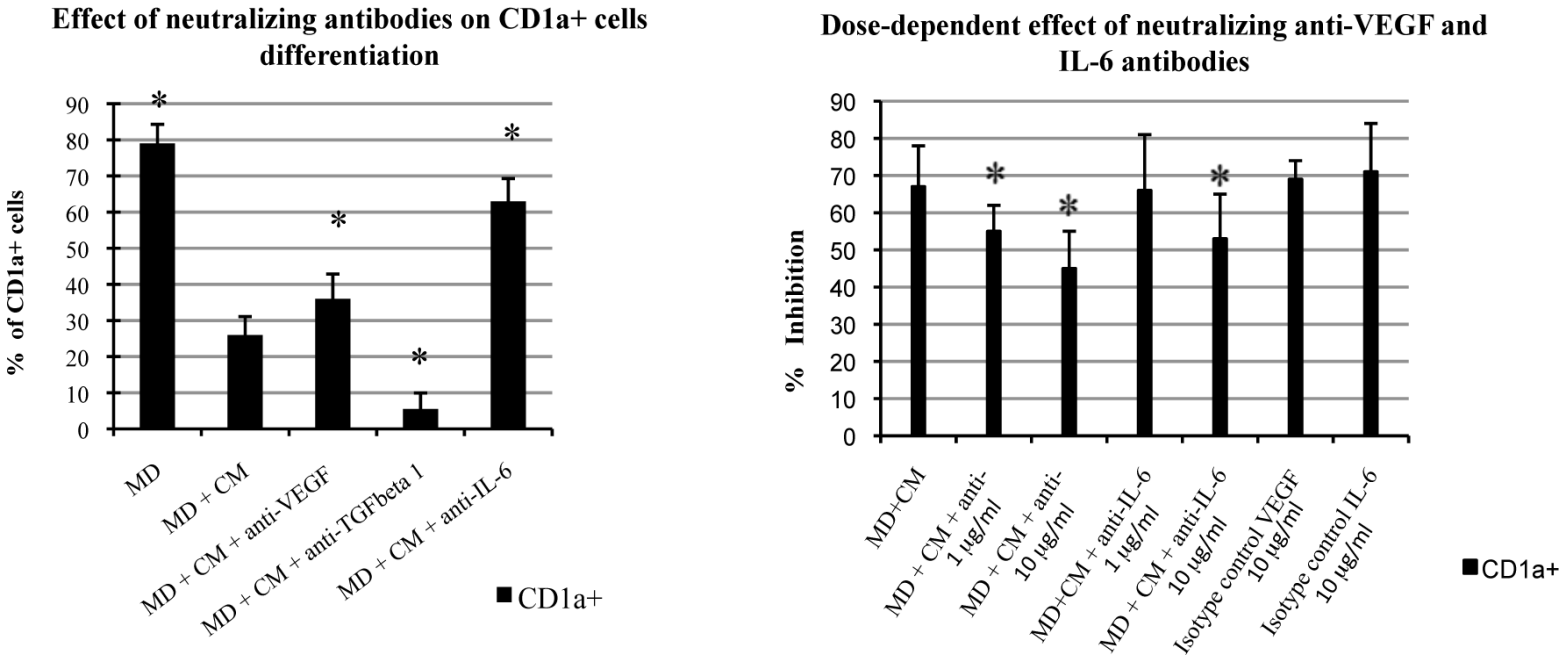
Antibodies against VEGF and IL-6 reduce the inhibitory effect of conditioned medium (CM) from GFs. A. Neutralizing antibodies against VEGF (10 µg/ml) and IL-6 (10 µg/ml) significantly reduced (P<0.05) the inhibition of CD1a+ monocytes-derived cells by CM from GFs. In contrast, neutralizing antibodies against TGFβ1 significantly increased (P<0.05) this inhibitory effect. B. The effect of the neutralizing antibodies against VEGF and IL-6 was dose-dependent. The results reported are means ±SD for four independent experiments in duplicate. *Significant difference to the value for MD+CM (P<0.05).

We investigated the contributions of IL-6, VEGF and TGFβ1 implicated by the ELISA results, in the differentiation of monocyte-derived DCs by using antibodies to neutralize these factors in CM from GFs (Figure 4). Neutralizing antibodies against IL-6 and VEGF significantly reduced (P<0.05) the inhibitory effect of CM (five independent experiments in duplicate with different CM and monocytes from different donors) whereas neutralizing antibodies against TGFβ1 significantly increased (P<0.05) the inhibitory effect (Fig. 4A). The effects of neutralizing antibodies against VEGF and IL-6 were dose-dependent (Fig. 4B). Isotypic controls did not have any detectable effect on the differentiation of DCs. The differentiation of monocyte-derived DCs was not significantly affected by the addition of rVEGF (10 ng/mL, 20 ng/mL, 40 ng/mL or 80 ng/mL) to the culture medium (data not shown).

### Detection of IL-6 and its mRNA in Gingival Biopsies by ELISA and RT-PCR

To assess the relevance of our results, we tested for IL-6 in total protein extracts from gingival tissues by ELISA. IL-6 protein content was detected in all biopsies (mean concentration: 379.2±27 pg/mL). We confirmed the expression of IL-6 by quantitative RT-PCR using GAPDH as the internal standard gene: human IL6 mRNA was detected in all three total RNA preparations from biopsies (the mean level of IL-6 mRNA was 1.33±0.04; data not shown).

### Monocytes are Found Close to Fibroblasts in Gingival Tissue

**Figure 5:**
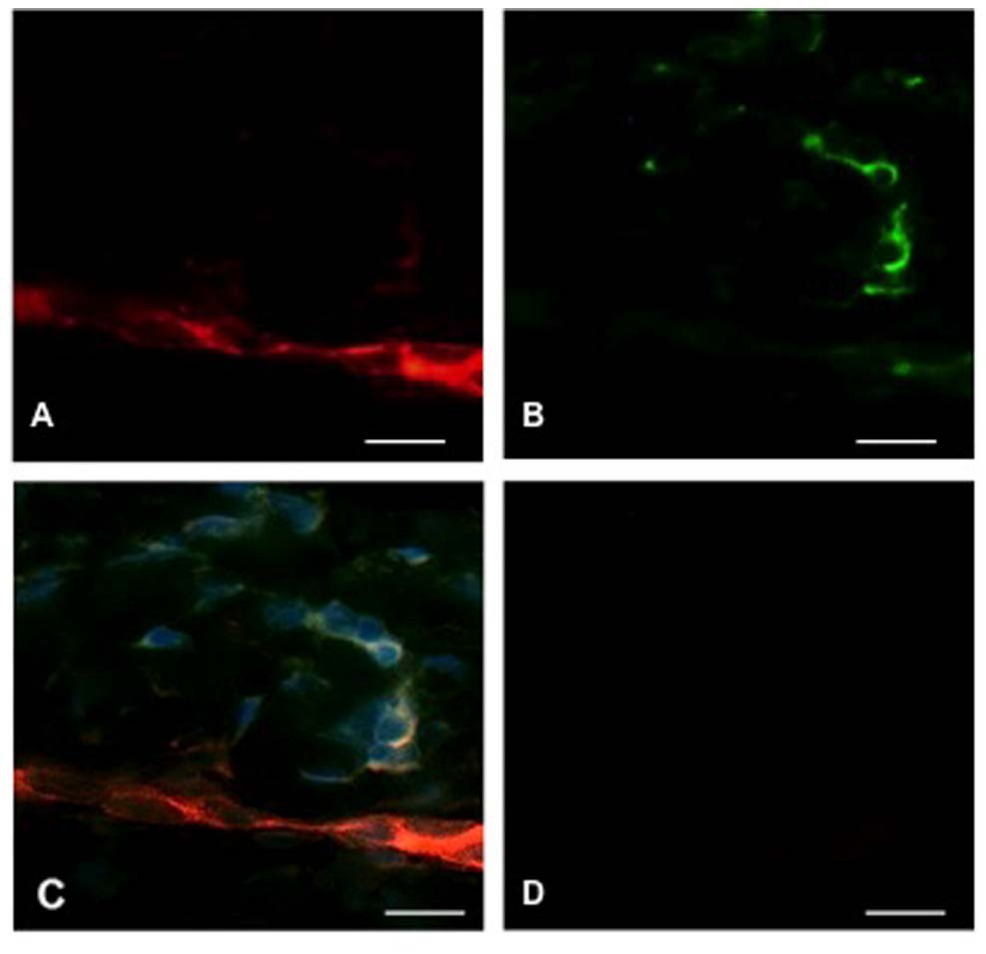
Co-localization of CD90^+^ gingival fibroblasts (GFs) and CD14^+^ monocytes in superficial gingival connective tissue. A. Numerous GFs are revealed as red fluorescence by PE-conjugated secondary antibody (magnification ×40). B. CD14^+^ monocytes are revealed as green fluorescence by FITC-conjugated secondary antibody (magnification ×40). C. DAPI staining with superposition of red and green fluorescence showing the close proximity of the two cell populations in the gingival tissue, consistent with communication between these cell types (magnification, ×40). D. Isotype-matched antibodies as negative controls (magnification ×40), scale bar: 50 µm.

To demonstrate that the interaction between GFs and DCs is relevant *in vivo*, we tested for co-localization of fibroblasts and monocytes in gingival tissue. Immunofluorescence labeling revealed CD90^+^ GFs and CD14^+^ monocytes in close proximity in superficial gingival connective tissue (Figure 5).

## Discussion

We report that gingival fibroblasts (GFs) inhibit the differentiation of CD1a^+^ monocyte-derived dendritic cells (DCs). By using Transwell® equipment allowing co-culture of monocytes and fibroblasts without contact, as well as various conditioned media (CM) from GFs, we show that this effect is mediated by soluble factors. The inhibition was associated with a lower stimulatory activity in MLR for DCs generated with CM than controls. We demonstrated a dose-dependent inhibitory effect of GFs, and the inhibition of the differentiation of CD1a^+^ monocyte-derived DCs was observed using four, independent, CM from GFs (Table 1). The effect of CM was thus reproducible and donor independent. IL-6 and VEGF were produced by GFs, and neutralizing antibodies specific for these factors partially prevented the inhibitory effect of CM from GFs. IL-6 and M-CSF can inhibit the differentiation of DCs [5]–[7]; thus, like dermal fibroblasts, GFs release IL-6 which up-regulates the expression of M-CSF receptors on monocytes allowing the utilization of their autocrine M-CSF leading to their differentiation to macrophages rather than DCs. VEGF has large effects on the differentiation of several hematopoietic lineages [17], [18]. We observed that VEGF released by GFs completely disappeared from the medium in the presence of monocytes, which express the VEGFR1/Flt1 receptor implicated in monocyte migration [19]. This findings is in agreement with a previous study suggesting that DCs regulate the expression of VEGF by their capacity to clear VEGF via Flt-1 [20].

Dendritic cells are important in the priming of the immune response. The blockade of their differentiation by IL-6 and VEGF may lead to a state of tolerance associated with an increase of regulatory T cells with suppressive activity [21], [22]. VEGF may also potentiate immunosuppression via direct amplification of regulatory T cells [23].

Our findings suggesting that GFs may contribute to sustaining an immunosuppressive microenvironment are coherent with recent data showing that human colonic fibroblasts promote the expansion of regulatory T cells [24]. Although VEGF has also been reported to inhibit the maturation of dendritic cells [17], [18], [25], we did not observe any effect of CM from GFs on the inhibition of this process; this may have been due to the presence of other factors present in the CM and counterbalancing the effect of VEGF. We show that neutralizing antibodies against TGFβ1 also produced by GFs, increased the inhibitory effect of CM from GFs on the differentiation of DCs. This is consistent with previous studies showing that TGFβ1 accelerates dendritic cell differentiation from common DC progenitors and directs subset specification toward conventional DCs [26], [27].

Our findings for the inhibitory activity of GFs are in line with, and extend, previous work showing that GFs suppress allogeneic peripheral blood mononuclear cell (PBMNC) proliferation independently of any cell-cell contact [28]. Dermal fibroblasts are able to regulate T cell development and survival [29], [30] and to inhibit allogeneic T cell activation by autologously derived cutaneous antigen-presenting cells (APCs) [31]. Other stromal cells in the oral mucosa exhibit immunosuppressive activity: a recent study [32] revealed that oral mucosal lamina propria progenitor cells (OMLP-PCs) are potently immunosuppressive in a dose-independent manner. Also, human periodontal ligament stem cells (PDLSCs) display cell surface marker characteristics and a differentiation potential similar to bone marrow stromal stem cells (BMSSCs), and inhibited peripheral blood mononuclear cell (PBMNC) proliferation stimulated with mitogen or in an allogeneic mixed lymphocyte reaction (MLR) [28]. Mesenchymal stem cells, which are considered to be phenotypically similar to GFs [33], can influence the early stages of DC differentiation [34]–[38] and have pleiotropic immunoregulatory functions including immunosuppressive activities. This has led to their therapeutic use in humans [28], [39]–[42]. The role of GFs in the differentiation of DCs is not similar to that of MSCs. Indeed, depending on studies, the supernatants of MSCs inhibit [7] or do not inhibit [37] the differentiation of bone marrow progenitors into DCs via the production of IL-6. In our experimental model, unlike previous studies using conditioned media from MSCs [37], CM from GFs did not affect the maturation of DCs.

The human gingiva contains CD1a^+^ Langerhans cells and connective tissue DCs directly implicated in the initiation and maintenance of inflammatory periodontal disease triggered by bacterial plaque and its metabolic products [43]. DCs are also essential for inducing and maintaining mucosal immune homeostasis associated with harmless commensal bacteria, and for causing antigen-specific unresponsiveness or tolerance by several mechanisms [44] including induction of regulatory T cells [45]. The cytokine microenvironment of monocytes and DCs in the periphery is critical to the type of adaptive immune response (stimulatory or inhibitory) that DCs induce. Our findings are relevant to the local microenvironment of the gum, as we demonstrated the co-localization of CD14^+^ monocytes and GFs (figure 5) which may participate in the regulation of local inflammation and homeostasis. This close proximity may facilitate the modulation of the differentiation of CD1a^+^ Langerhans cells by soluble mediators released by GFs before their migration towards the epithelium. This interaction would favor a more tolerogenic DC phenotype.

Our various findings demonstrate that VEGF and IL-6 are both involved in the inhibition of DC differentiation mediated by GFs. Immunomodulation seems to be a general property of stromal cells, including MSCs and fibroblasts. MSCs are already used in cell therapy to inhibit inflammatory reactions [46]. GFs are easily accessible and cultured, and are a possible alternative source of stromal cells for cell therapy. We show that in addition to the potential of GFs in the protection and repair of the extra-cellular matrix [47], GFs have immunomodulatory effects such that they have two activities that are potentially valuable for cell therapy of inflammatory diseases.
